# Draft Genome Sequence of Purine-Degrading *Clostridium cylindrosporum* HC-1 (DSM 605)

**DOI:** 10.1128/genomeA.00917-15

**Published:** 2015-08-13

**Authors:** Anja Poehlein, José D. Montoya Solano, Frank R. Bengelsdorf, Bettina Schiel-Bengelsdorf, Rolf Daniel, Peter Dürre

**Affiliations:** a Genomic and Applied Microbiology & Göttingen Genomics Laboratory, Georg-August University Göttingen, Göttingen, Germany; b Institut für Mikrobiologie und Biotechnologie, Universität Ulm, Ulm, Germany

## Abstract

Here, we report the draft genome sequence of *Clostridium cylindrosporum* HC-1, a purine- and glycine-fermenting anaerobe, which uses selenoprotein glycine reductase for substrate degradation. The genome consists of a single chromosome (2.72 Mb) and a circular plasmid (14.4 kb).

## GENOME ANNOUNCEMENT

Anaerobic purine degradation was first discovered by Liebert when he isolated *Bacillus acidiurici* that decomposed uric acid to acetate, carbon dioxide, and ammonia (1). Later, Barker and Beck isolated and described the purine-degrading *Clostridium acidiurici* (later *C. acidurici*) and *C. cylindrosporum* from soil throughout California and from Provo, Utah (2, 3). *C. acidurici* is most likely Liebert’s *B. acidiurici* (4). Differentiation between the two species was first based on morphological features and the production of glycine and formate besides acetate. Other features proposed were amino acid composition and immunochemistry of ferredoxin and formyltetrahydrofolate synthetase (5), trace element requirement for optimal formate dehydrogenase activity (6), and nutritional requirements (6, 7). As routine identification methods did not allow an easy distinction, *C. cylindrosporum* was removed from the approved lists of bacterial names (8) and the original name was only later revived (9). Finally, DNA-DNA hybridization allowed a clear-cut distinction between strains of *C. acidurici* and *C. cylindrosporum* (10). *C. acidurici* was recently reclassified into the new genus *Gottschalkia* (11). The genome sequence of *G. acidurici* has already been reported (12), and the *C. cylindrosporum* sequence reported here complements these data.

Chromosomal DNA was isolated using the MasterPure complete DNA purification kit (Epicentre, Madison, WI, USA). Illumina shotgun libraries were generated from the extracted DNA according to the protocol of the manufacturer. Sequencing was performed using the Genome Analyzer IIx (Illumina, San Diego, CA) resulting in 4,303,040 112-bp paired-end reads.

The *de novo* assembly performed with the SPAdes genome assembler software 3.5.0 (13) resulted in 28 contigs (>500 bp) and an average coverage of 176-fold. The genome of *C. cylindrosporum* consists of a chromosome (2.72 Mb) and a plasmid (14.4 kb) with an overall G+C content of 31.46%. Automatic gene prediction was performed using the software tool Prodigal (14). Genes coding for rRNA and tRNA were identified using RNAmmer (15) and tRNAscan (16), respectively. The Integrated Microbial Genomes-Expert Review (IMG-ER) system (17) was used for automatic annotation, which was subsequently manually curated by using the Swiss-Prot, TrEMBL, and InterPro databases (18). The genome harbored 9 rRNA genes, 65 tRNA genes, 1,879 protein-coding genes with predicted functions, and 743 genes coding for hypothetical proteins.

Genome analysis also revealed the presence of genes encoding a glycine reductase comparable to those identified in *Eubacterium acidaminophilum* (19) and *Sporomusa ovata* (20), or *C. litorale* (21) including the *grdX* gene, which is always located upstream of the glycine reductase operon. The function of this protein is not clear yet, but it may either have a regulatory function or it is involved in the enzymatic glycine reduction. We therefore propose to annotate this gene as glycine reductase-associated protein GrdX. This is clearly different than *G. acidurici*, which was found not to harbor glycine reductase genes (12). Genome inspection also showed that genes coding for the carbonyl branch of the Wood-Ljungdahl pathway are not present.

### Nucleotide sequence accession numbers.

This whole-genome shotgun project has been deposited at DDBJ/EMBL/GenBank under the accession no. LFVU00000000. The version described in this paper is version LFVU01000000.
